# The application of basic SMAS rhytidectomy and comprehensive SMAS rhytidectomy

**DOI:** 10.1097/MD.0000000000040126

**Published:** 2024-10-25

**Authors:** Yin-Jie Ao, Yan Yi, Yun-Fei Nie, Guo-Hui Wu

**Affiliations:** a The Affliated Eye Hospital, Jiangxi Medical College, Nanchang University, Jiangxi Province, PR China; b Zhengxing Stomatalogical Hospital, Yichun City, Jiangxi Province, PR China; c ZJY Plastic and Aesthetic Clinic, Guangzhou City, Guangdong Province, PR China.

**Keywords:** basic, comprehensive, SMAS rhytidectomy

## Abstract

Rhytidectomy has made great progress in the past 50 years, especially after the proposition of the concept of superficial musculoaponeurotic system (SMAS). Our previous research had classified SMAS rhytidectomy into basic SMAS rhytidectomy (B-SMAS) and comprehensive SMAS rhytidectomy (C-SMAS) according to whether the treatment of SMAS aponeurosis is combined with retaining ligament, fat pad, mimetic muscles, etc. The purpose of this paper is to compare the outcomes of the 2 methods. Through multicenter collaborative research, 21 cases of B-SMAS and 18 cases of C-SMAS were collected. Photographs were taken pre- and post-operation for each patient, and the facial width of the lateral canthus level (upper face), the alar base level (middle face), and the oral commissure level (lower face) were measured. We compared the statistics of upper, middle, and lower face pre- and post-B-SMAS and C-SMAS to assess the ramifications of each operation. We used preoperative measurements minus corresponding postoperative measurements of specific operation to assess which position would acquire superior improvements. We also recruited 3 professional plastic clinicians to rate the ramifications of specific positions and approaches after pre- and post-photo comparing. Through comprehensive analyzing, we compared the improvements between B-SMAS and C-SMAS to assess which method is more beneficial concerning facial rejuvenation. Both B-SMAS and C-SMAS would acquire optimal changing concerning facial width in middle and lower face after operation (*P* < .05). Two methods all harvested better width changing effects in middle and lower face than upper face (*P* < .05). However, C-SMAS acquired better effects in middle and lower face than B-SMAS (*P* < .05). Both B-SMAS and C-SMAS are beneficial to facial rejuvenation. The effects in middle and lower face are better than upper face. In addition, C-SMAS may be more effective than B-SMAS.

## 1. Introduction: development of SMAS rhytidectomy

In 1974, by elevating the platysma flap and the facial superficial aponeurosis together, Skoog completed the first rhytidectomy that did not aim at elevating skin alone, and then embarked the era of superficial musculoaponeurotic system (SMAS) rhytidectomy.^[1]^ Subsequently, with the introduction of the concepts such as SMAS, facial retaining ligament, and facial nerve risky zone, scholars have further enhanced their understandings of SMAS rhytidectomy.^[2–5]^ Simultaneously, surgical methods have also progressed significantly. Hamra and Barton et al proposed the concept of deep plane composite SMAS rhytidectomy by elevating SMAS and skin composite tissue flap together.^[6,7]^ Owsley, O’Connell, Aston, and Stuzin et al totally dissected SMAS and elevated it alone, even though the method enhanced possibility of SMAS impairment, however, the utmost extending of SMAS would acquire.^[8–13]^ Baker and Tonnard et al proposed a coronal small incision for microinvasive frontal SMAS elevation.^[14,15]^ O’Connell et al did not dissect SMAS, only buried threads on the surface to elevated it in dual directions.^[16]^

We summarized various approaches of SMAS rhytidectomy and divided them into 2 categories. Comprehensive SMAS rhytidectomy (C-SMAS), which dissected SMAS extensively as well as including one or more following dispositions such as one (3 cases) or more retaining ligaments cutting and reconstructing (15 cases), SMAS cutting (18 cases) and reanchoring (18 cases), one (3 cases) or more fat pad suspension (15 cases) and dispositions of mimetic muscles (14 cases).^[17]^ Basic SMAS rhytidectomy (B-SMAS), which dissected SMAS locally (15 cases) or not (6 cases), and implemented at least one following disposition such as SMAS plication (7 cases), imbrication (8 cases), thread sculpturing (4 cases) or limited removing without managing the retaining ligaments or subcutaneous tissues (2 cases).^[17]^ We aim to provide objective and subjective analyzing of statistics, and compare ramifications between B-SMAS and C-SMAC in facial rejuvenation.

## 2. Methodology

In this study, we collected clinical cases through multicenter cooperation, and measured the facial width of lateral canthus level (upper face), alar base level (middle face), and the oral commissure level (lower face) for each case pre- and post-operation.^[18–20]^ We also recruited 3 professional plastic clinicians to rate the ramifications of specific positions and approaches after pre- and post-photo comparing.^[21]^ Through comprehensive analyzing, we accumulated statistics objectively and compared the operational effects of B-SMAS and C-SMAS. Finally, we try to put forward feasible suggestions concerning selection of specific surgical methods for different patients.

## 3. Patients and methods

### 3.1. Study sample

Our study was based on multi-institutional research. Cases accepted B-SMAS or C-SMAS between 2017 and 2022 at the 2nd Hospital Affiliated to Yichun University (Yichun, China), Zhengxing Stomatological Hospital (Yichun, China), Boyiangfang Plastic and Aesthetic Clinic (Guangzhou, China), Ophthalmology Hospital affiliated to Nanchang University (Nanchang, China). Before enrollment into the study, participants were informed about the aim and the scope of this study and provided written informed consent for the use of their research-related and demographic data. The study was approved by the institutional review board of The Affliated Eye Hospital, Jiangxi Medical College, Nanchang University (Protocol number YLS20240439).

### 3.2. Statistics accumulation

**Figure 1.**
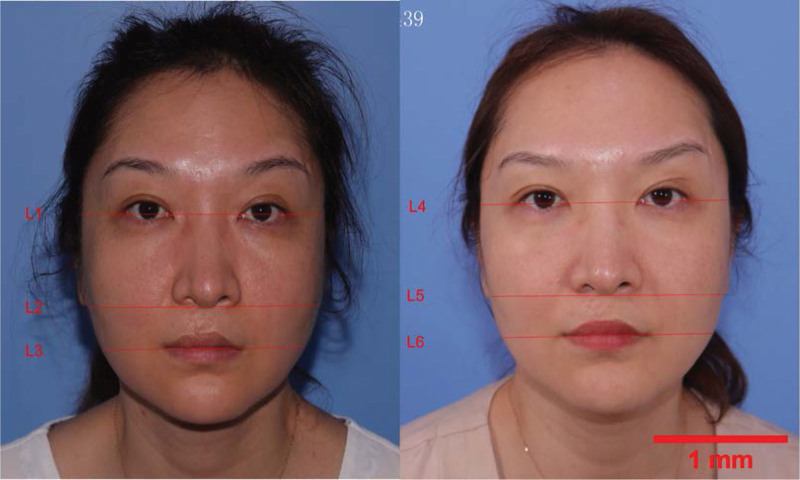
B-SMAS. Left: before operation, L1: facial width of the lateral canthus level (upper face), L2: facial width of the alar base level (middle face), L3: facial width of the oral commissure level (lower face). Right: after operation, L4: facial width of the lateral canthus level (upper face), L5: facial width of the alar base level (middle face), L6: facial width of the oral commissure level (lower face). B-SMAS = basic SMAS rhytidectomy.

**Figure 2.**
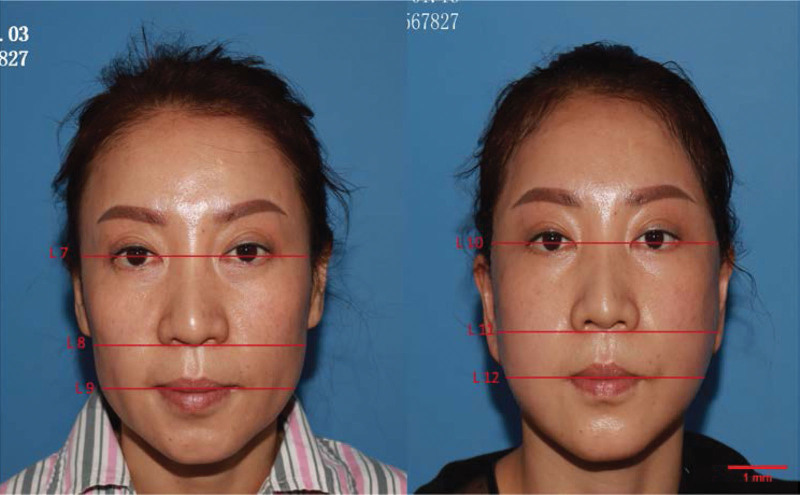
C-SMAS. Left: before operation, L7: facial width of the lateral canthus level (upper face), L8: facial width of the alar base level (middle face), L9: facial width of the oral commissure level (lower face). Right: after operation, L10: facial width of the lateral canthus level (upper face), L11: facial width of the alar base level (middle face), L12: facial width of the oral commissure level (lower face). C-SMAS = comprehensive SMAS rhytidectomy.

We randomly assigned patients to receive B-SMAS and C-SMAS based on their own surgical preferences. Photographs were taken pre- and post-operation for each patient, and the facial width of the lateral canthus level (upper face), the alar base level (middle face) and the oral commissure level (lower face) were measured. We accumulated the statistics of B-SMAS (Fig. 1) and C-SMAS (Fig. 2) after used preoperative measurements minus corresponding post-operative measurements. We also recruited 3 professional plastic clinicians to rate the ramifications of specific positions and approaches after pre- and post-photo comparing. The rated methods were very satisfied 3 points, satisfied 2 points, no visible improvement 1 point. The post operational data were accumulated at the 3 months after removed the stitches. Through literature research, we found that the 3 months can be used as the evaluative time to assess postoperative effects.^[20,22–32]^

### 3.3. Statistics analysis

We compared the statistics of upper, middle and lower face pre- and post-B-SMAS and C-SMAS to assess the ramifications of each operation. We used preoperative measurements minus corresponding postoperative measurements of specific operation to assess which position would acquire superior improvements. We also analyzed points of clinicians between B-SMAS and C-SMAS, as well as different positions in the same operation. After subjective pointing and objective measuring, we tried to figure out which method is more beneficial concerning facial rejuvenation. All patients were documented through images with frontal views of the face, and frontal views using a Nikon D80 camera and twin flash Canfield device (Canfield Connector, OH). Image J (NIH, Bethesda, MD) was used for photo measuring. SPSS Version 26 (IBM Corp, Armonk, NY) was used for *t* test and results were considered statistically significant when *P* < .05. Pearson *r* test were used to analyze practical significance of the effect. 0.8–1.0 very strong correlation, 0.6–0.8 strong correlation, 0.4–0.6 moderate correlation, 0.2–0.4 weak correlation, 0–0.2 no correlation. Statistic graphics were delineated via Graph Pad (Graph Pad Corp, San Diego, CA).

### 3.4. Complications

No severe complications were observed, tiny infection of stitches and temporary swelling could totally recover without any treatments.

## 4. Results

**Figure 3.**
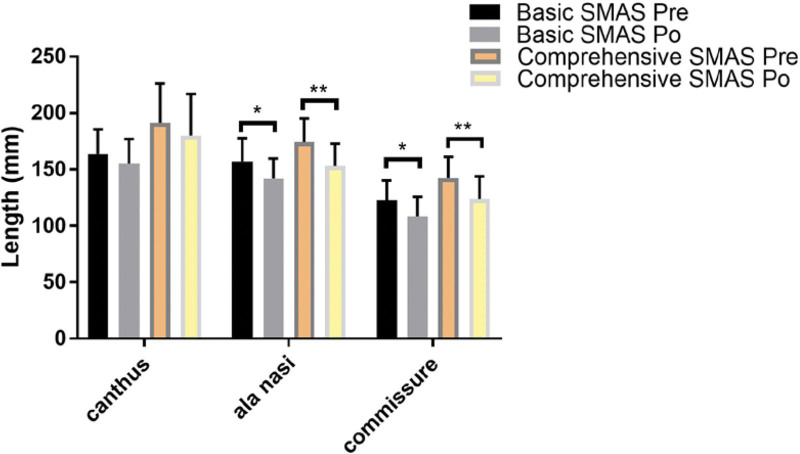
In measuring assessing, both B-SMAS and C-SMAS would acquire optimal changing concerning facial width in middle and lower face after operation (*P* < .05). (“*” means *P* < .05, “**” means *P* < .01). B-SMAS = basic SMAS rhytidectomy, C-SMAS = comprehensive SMAS rhytidectomy.

**Figure 4.**
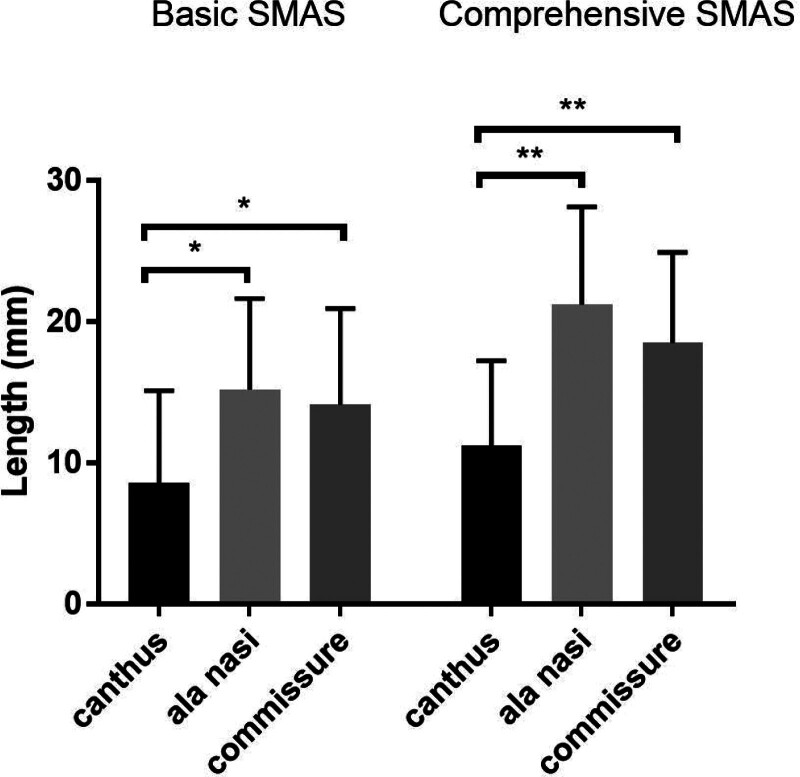
In measuring assessing, 2 methods all harvested better width changing effects in middle and lower face than upper face (*P* < .05). (“*” means *P* < .05, “**” means *P* < .01).

**Figure 5.**
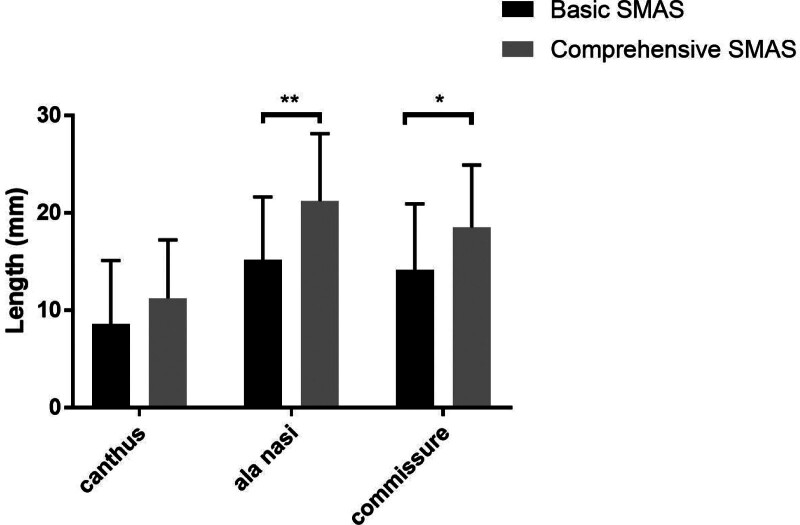
In measuring assessing, C-SMAS acquired better effects in middle and lower face than B-SMAS (*P* < .05). (“*” means *P* < .05, “**” means *P* < .01). C-SMAS = comprehensive SMAS rhytidectomy.

**Figure 6.**
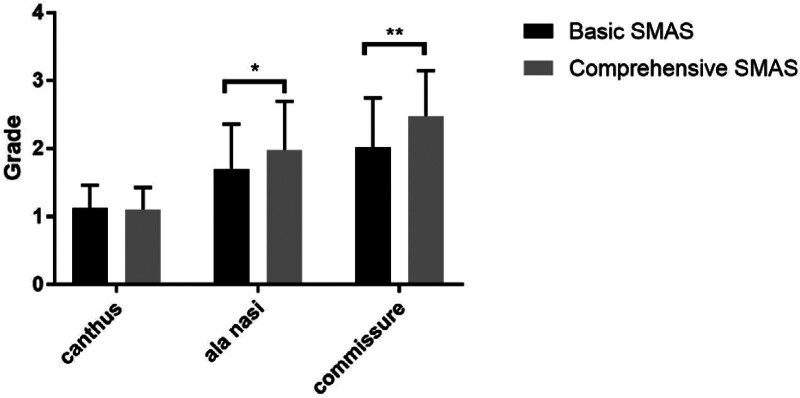
In pointing assessing, C-SMAS acquired better effects in middle and lower face than B-SMAS (*P* < .05). (“*” means *P* < .05, “**” means *P* < .01). C-SMAS = comprehensive SMAS rhytidectomy.

**Figure 7.**
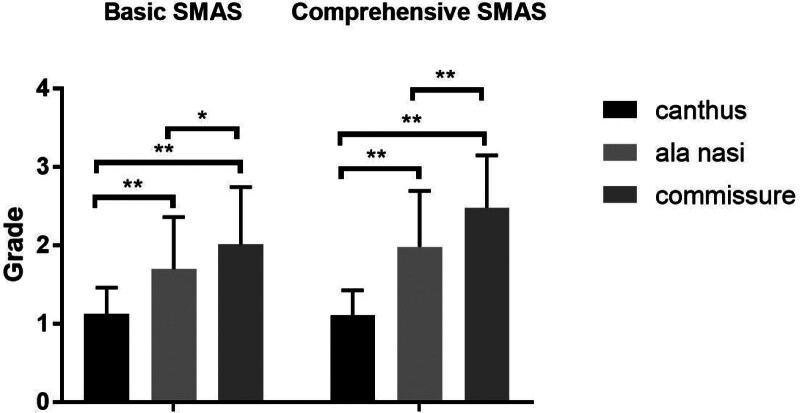
In pointing assessing, 2 methods all harvested better width changing effects in upper, middle and lower face than upper face (*P* < .05). (“*” means *P* < .05, “**” means *P* < .01).

**Figure 8.**
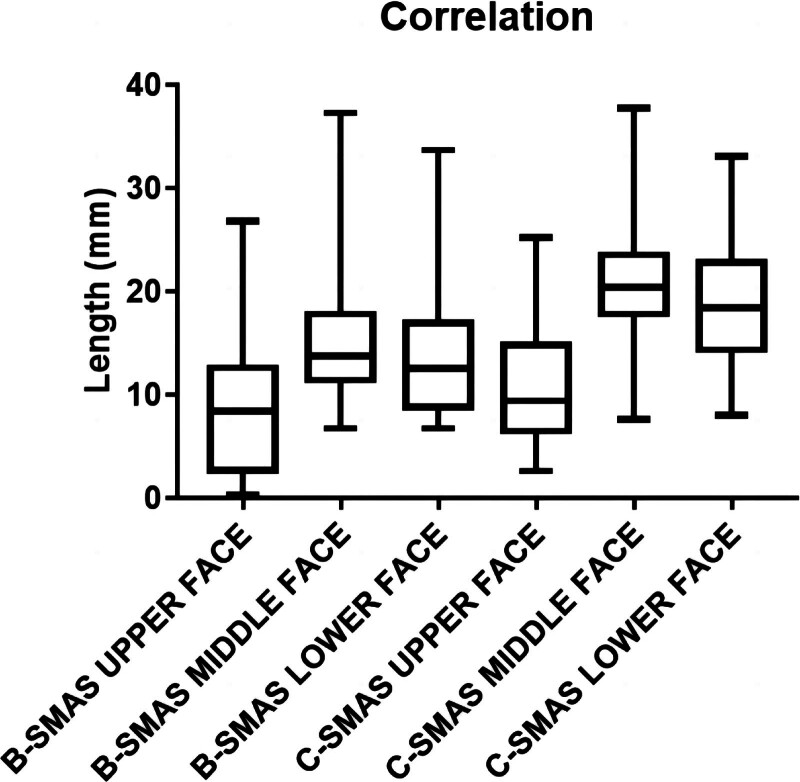
Pearson *r* test were used to analyze practical significance of the effect.

A total of 39 cases were selected into our experiment: B-SMAS 21 cases (1 male, 20 female), with mean age of 41 ± 8.733. C-SMAS 18 cases (1 male, 17 female), with mean age of 39.688 ± 5.896. In measuring assessing (Table 1), Pre-operation: B-SMAS, upper face 163.635 ± 21.813, n = 21, middle face 157.234 ± 20.573, n = 21, lower face 122.556 ± 17.762, n = 21; C-SMAS, upper face 191.424 ± 34.668, n = 18, middle face 174.238 ± 20.872, n = 18, lower face 142.439 ± 18.733, n = 18. Post-operation: B-SMAS, upper face 155.042 ± 22.026, n = 21, middle face 142.045 ± 17.586, n = 21, lower face 108.425 ± 17.574, n = 21; C-SMAS, upper face 180.197 ± 36.790, n = 18, middle face 153.017 ± 19.889, n = 18, lower face 123.931 ± 19.867, n = 18. Pre-operation minus post-operation: B-SMAS, upper face 8.593 ± 6.517, n = 21, middle face 15.189 ± 6.453, n = 21, lower face 14.131 ± 6.8, n = 21; C-SMAS, upper face 11.227 ± 6.021, n = 18, middle face 21.221 ± 6.928, n = 18, lower face 18.508 ± 6.394, n = 18. In pointing assessing (Table 2), B-SMAS, upper face 1.127 ± 0.336, n = 63, middle face 1.698 ± 0.663, n = 63, lower face 2.016 ± 0.729, n = 63; C-SMAS, upper face 1.111 ± 0.317, n = 54, middle face 1.981 ± 0.714, n = 54, lower face 2.481 ± 0.666, n = 54. Both B-SMAS and C-SMAS acquired optimal changing concerning facial width in middle and lower face after operation (*P* < .05) (Fig. 3). Two methods all harvested better width changing effects in middle and lower face than upper face (*P* < .05) (Figs. 4 and 7). However, C-SMAS acquired better effects in middle and lower face than B-SMAS (*P* < .05) (Figs. 5 and 6). In measuring analyzing, no significant differences happened when comparing improvement between middle and lower face. In pointing analyzing, significant differences happened concerning the outcomes mentioned above (Figs. 4 and 7). Upper face lift has correlation with middle and lower face lift respectively both in B-SMAS and C-SMAS (Fig. 8). Compared with Western countries, the age of Chinese people undergoing rhytidectomy is relatively early, between 40 and 50 years old. Therefore, the amount of tissue removal is relatively small, the operation focuses on solving tissue sagging, and the overall contour can be basically maintained, so we believe that the patient cannot change the ID photo.

**Table 1:** Measuring assessing.

International unit: mm		B-SMAS (mean ± SD，N = 21)	C-SMAS (mean ± SD，N = 18)
Pre-operation	Upper face	163.635 ± 21.813	191.424 ± 34.668
	Middle face	157.234 ± 20.573	174.238 ± 20.872
	Lower face	122.556 ± 17.762	142.439 ± 18.733
Po-operation	Upper face	155.042 ± 22.026	180.197 ± 36.790
	Middle face	142.045 ± 17.586	153.017 ± 19.889
	Lower face	108.425 ± 17.574	123.931 ± 19.867
Pre-operation minus post-operation	Upper face	8.593 ± 6.517	11.227 ± 6.021
	Middle face	15.189 ± 6.453	21.221 ± 6.928**
	Lower face	14.131 ± 6.8	18.508 ± 6.394*

B-SMAS = basic SMAS rhytidectomy, C-SMAS = comprehensive SMAS rhytidectomy.

* *P* < .05.

** *P* < .01.

**Table 2:** Pointing assessing.

International unit: Point	B-SMAS (mean ± SD，N = 63)	C-SMAS (mean ± SD，N = 54)
Upper face	1.127 ± 0.336	1.111 ± 0.317
Middle face	1.698 ± 0.663	1.981 ± 0.714**
Lower face	2.016 ± 0.729	2.481 ± 0.666*

B-SMAS = basic SMAS rhytidectomy, C-SMAS = comprehensive SMAS rhytidectomy.

* *P* < .05.

** *P* < .01.

## 5. Discussion

With the development of economy, comprehensive treatments for facial rejuvenation have made great progress.^[33]^ At present, the main methods are photoelectric, noninvasive injections, thread carving and surgery.^[33]^ However, facial aging is a multi-factorial process and concerning all facial tissues, such as skeletons, muscles, ligaments, fat and skin.^[34]^ Thus, different approaches and modalities have their own indications.^[34]^ When severe saggy happening or the patient had already accepted various noninvasive or photoelectric methods but still cannot acquire plausible ramifications.^[17]^ Rhytidectomy may become the unique method. In 1974, through elevating platysma and facial superficial musculoaponeurotic fascia (SMAS) together, Skoog completed the first rhytidectomy without elevating skin uniquely, which also embarked the era of SMAS rhytidectomy.^[1]^ Subsequently, with the proclamation of concepts such as SMAS, facial retaining ligaments and danger zone of facial nerve, scholars also enhanced understandings of SMAS rhytidectomy.^[35–37]^ To our knowledge, even though the title of this operation is dubbed as rhytidectomy, SMAS rhytidectomy may not be aimed at specific wrinkle, and the primary purpose of this operation is to solve a facial sagging problem and then achieve facial rejuvenation.

With enhancing the range of SMAS undermining in the operation, the possibility of facial nerve impairment also increasing.^[38]^ Especially in extended deep plane SMAS rhytidectomy, the undermining range will reach nasolabial fold.^[39]^ According to Sadati, it is not profitable to undermine SMAS extensively if the process enhanced the possibility of facial nerve impairment.^[40]^ As we all know, the branches of facial nerve located superficially when proceed medially.^[38]^ Simultaneously, the primary zygomatic retaining ligament, the masseteric retaining ligament and the submandibular retaining ligament constituted a curving line, which divided the face into relative moveable area laterally and relative static area medially.^[36]^ Therefore, the dominating ramifications of elevating may acquire when undermining area surpass the curving line with the retaining ligaments be transected, and further SMAS undermining may not beneficial enough for it may enhanced the possibility of facial nerve impairment severely.^[41]^ And then, more and more scholars modified the operation, they implemented thread lifting in the SMAS or plicated or imbricated the fascia without any undermining.^[42,43]^ Wu limited undermined SMAS, the process will be ceased if nasolabial fold become shallower than before.^[35]^ All of the approaches mentioned above can only attained relative limited elevating effects for they do not transect the retaining ligaments.^[44]^ However, the nominations of the operations varied.

We tried to classify SMAS rhytidectomy into B-SMAS and C-SMAS previously.^[17]^ If SMAS undermining combined with at least one following item such as retaining ligament transecting and reanchoring, fat pad excision and reanchoring, mimetic muscle excision, we nominated the operation as C-SMAS.^[17]^ Other operations such as thread elevating SMAS,plication or imbrication of SMAS, limited undermining SMAS with localized excision, we nominated them as B-SMAS.^[17]^ B-SMAS is suitable for patients with moderate facial sagging, who had already accepted filler injection or thread lifting and still can’t reach its purpose. C-SMAS is suitable for severe sagging patients with apparent nasolabial sulcus or tear trough, blurring jawline, saggy jowl, deep marionette. In this research, we divided patients into B-SMAS group and C-SMAS group. According to our research, both B-SMAS and C-SMAS acquired plausible ramifications of facial rejuvenation, especially in middle and lower face. In addition, C-SMAS may acquire more preferable outcomes than B-SMAS in middle and lower facial rejuvenation. Other research also analyzed effects of different SMAS rhytidectomy previously, but most of them relied on subjective scoring system or rating scales, which may lack of objectivity.^[16,45–47]^ Through documents analyzing, we measured specific facial length pre- and post- operation and accumulated statistics.^[18]^ Simultaneously, we also applied pointing method to assess the outcomes. We found that in measuring analyzing, no significant differences happened when comparing improvement between middle and lower face. In pointing analyzing, significant differences happened concerning the outcomes mentioned above. This may reflect that objective assessing will be more reliable than subjective assessing, especially to specific module required precise assessing.

We still have shortcomings in this study. Recently, facial rejuvenation projects in the Chinese cosmetic market are dominated by microsurgery and photoelectric therapy. And then, cases are not abundant enough because most potential patients lack comprehensive understandings concerning SMAS rhytidectomy. Simultaneously, compliance of patients in Chinese private stated medical institutions is difficult to access, especially for long-lasting follow up. We removed the stitches at the seventh day post operation and then measured facial lengths in order to coincide the measuring time. Obviously, if long term and periodic follow up is easily accessible, the intermittent assessing will be more convincing.

## 6. Conclusion

After objective and subjective analyzing of statistics, we comprehensively compared ramifications between B-SMAS and C-SMAC in facial rejuvenation. We hope to provide plausible considerations concerning alternative of specific operational method to clinicians in the future.

## Author contributions

**Conceptualization:** Yun-Fei Nie, Guohui Wu.

**Data curation:** Yun-Fei Nie.

**Formal analysis:** Yun-Fei Nie.

**Resources:** Yan Yi.

**Writing – original draft:** Yinjie Ao.

**Writing – review & editing:** Yinjie Ao.
